# Antimicrobial Nanostructured Coatings: A Gas Phase Deposition and Magnetron Sputtering Perspective

**DOI:** 10.3390/ma13030784

**Published:** 2020-02-08

**Authors:** Giulio Benetti, Emanuele Cavaliere, Francesco Banfi, Luca Gavioli

**Affiliations:** 1 Interdisciplinary Laboratories for Advanced Materials Physics (i-LAMP) and Dipartimento di Matematica e Fisica, Università Cattolica del Sacro Cuore, Via Musei 41, 25121 Brescia, Italy; giulio.benetti@gmail.com (G.B.); Emanuele.Cavaliere@unicatt.it (E.C.); 2 Department of Pathology and Diagnostics–Medical Physics Unit, University Hospital of Verona, P.le Stefani 1, 37126 Verona, Italy; 3 Université de Lyon, CNRS, Université Claude Bernard Lyon 1, Institut Lumière Matière, F-69622 Villeurbanne, France; francesco.banfi@univ-lyon1.fr

**Keywords:** antimicrobial coatings, single and multi-element nanoparticles, metals, oxides, supersonic beams, mechanical properties, granular materials, functional materials, magnetron sputtering, clusters

## Abstract

Counteracting the spreading of multi-drug-resistant pathogens, taking place through surface-mediated cross-contamination, is amongst the higher priorities in public health policies. For these reason an appropriate design of antimicrobial nanostructured coatings may allow to exploit different antimicrobial mechanisms pathways, to be specifically activated by tailoring the coatings composition and morphology. Furthermore, their mechanical properties are of the utmost importance in view of the antimicrobial surface durability. Indeed, the coating properties might be tuned differently according to the specific synthesis method. The present review focuses on nanoparticle based bactericidal coatings obtained via magneton-spattering and supersonic cluster beam deposition. The bacteria–NP interaction mechanisms are first reviewed, thus making clear the requirements that a nanoparticle-based film should meet in order to serve as a bactericidal coating. Paradigmatic examples of coatings, obtained by magnetron sputtering and supersonic cluster beam deposition, are discussed. The emphasis is on widening the bactericidal spectrum so as to be effective both against gram-positive and gram-negative bacteria, while ensuring a good adhesion to a variety of substrates and mechanical durability. It is discussed how this goal may be achieved combining different elements into the coating.

## 1. Introduction

Nanostructured materials (NMs) [1,2,3,4,5] represent an active area of research and a techno-economic sector in continuous expansion in many application domains, as shown by the more than 1400 review papers describing NMs applications that have appeared in the last three years. The technological importance of NMs is due to their tunable physicochemical characteristics, such as optical absorption [6,7,8], electrical conductivity [9,10], photothermal characteristics [11], optical [12,13] and photoacoustic sensing [14,15], surface enhanced Raman scattering effects [16,17], chemical state [18], and bactericidal functionalities [19,20,21]. In particular, the latter research sector has been driven by the interest in understanding the interactions and effects of nanoparticles (NPs) on living organisms [20,21,22,23,24,25,26,27], with prominent attention towards the wet synthesis of NMs and NPs and the study of their effect on cells and bacteria in solution. More recently, the effort of the scientific community in developing NMs-based antimicrobial agents has been stimulated by the emergence of multi-drug-resistant pathogens, posing a world-wide challenge with a large socio-economic impact [28,29]. In particular, a major issue is due to microbe spreading by 1) cross-contamination through infected surfaces in nosocomial environments [30,31] 2) biofilm formation on the surfaces of medical implants, such as dental or bone repair devices [32,33,34,35,36]. Infectious diseases caused by fungi, viruses, bacteria, and particularly by multidrug resistant bacteria has an estimated annual cost (direct and indirect) ranging from 6 to 60 billion US$ [37] in the US only. This is in addition to the limited number of new antibiotics successfully developed in the last few decades [38], due to the difficulty to find new antibacterial compounds with good pharmacological profiles and low toxicity and, from an economic point of view, to the higher interest of pharmaceutical companies in developing drugs for chronic conditions than for short-term treatments [39]. 

Where succesfull, the use of NMs could in principle avoid the problem of the antibiotic resistance mechanisms. As a matter of fact, because of the their mode of action, NMs should be less prone in promoting resistance in bacteria as compared to antibiotics [40]. Therefore a major goal is finding appropriate materials that are able to kill bacteria, such as metal-based NPs [40,41,42,43], but also obtaining functional nanostructured surfaces and thin film coatings [44,45] that can limit the spread of bacteria through surfaces. These former aspects increase the level of challenge, since the nanomaterial is required to have (1) microbicidal activity against a wide number of multi-drug-resistant Gram negative (GN) *and* Gram positive (GP) pathogens; (2) tunable mechanical properties and adhesion, to tailor the NPs release and the film durability in different conditions; and (3) cost-effective, environmental friendly production methods with high throughput. 

Furthermore, very recently, attention has also been focused on the problems that surfaces, with active-attack functions against bacteria, might suffer due to the accumulation of dead bacteria and organic debris [21]. Accumulation of organic debris on the surface might lead to shielding of the active killing material (in particular for chemically functionalized surfaces), thereby reducing the bactericidal efficacy. Some attempts have hence been made to combine passive-defense antibacterial surfaces, which reduce the rate of bacteria sticking, with the bactericidal functionality [46,47].

Such goals are strictly related to the ability of synthesizing NPs with the desired properties and also of assembling them at the nanoscale in such a way as to tailor the desired coating properties. The sought properties, however, might be very different, depending on he application target, i.e., on the type of surface where the antimicrobial agent has to be located, since topological and chemical characteristics of a surface determine the rate of microorganism adhesion [19,21,46,48]. In this respect, a large number of works have been devoted to understanding the interaction mechanism of the NP with living organisms. Although there are still important issues to be clarified, in particular with respect to the nanostructured coatings, there is an emergent consensus on the major processes underlying the antibacterial effects of NPs, as detailed in reference [40]. In a nutshell, these include NP penetration of the bacterial cell membrane, induction of intracellular antibacterial effects (e.g., interactions with DNA and proteins), and disruption of the bacterial cell membrane or generation of reactive oxygen species (ROS) [40]. All these processes are directly related to NPs’ size, composition and availability at the interaction interface between the coating and the bacteria.

Many different routes have been explored to synthesize NMs and NPs, from deposition of polymeric films [19,21,45,49,50,51] with incorporated bactericidal agents [21,52,53,54,55], or metallic or oxide films [2,21,56,57], to metal decoration of nanostructures [58,59,60]. The strategies are based on wet synthesis (spin-coating [61], sol–gel [57,61,62]), photochemical [52], biological [63,64,65], biotechnological [24] and physical methods (laser ablation [66], magnetron sputtering (MS) [2,44,67,68,69], gas phase beams [70,71]). Surprisingly, the literature available on nanostructured coatings obtained by physical methods is scarce.

This review is hence devoted to pointing out some aspects of antibacterial coatings, briefly describing what kind of materials can produce antibacterial effects, and then the issues related to the composition, mechanical properties and antimicrobial properties of coatings, focusing on some recent examples of coatings obtained with physical deposition techniques.

## 2. Interaction Mechanisms between Bacteria and NMs

**Figure 1:**
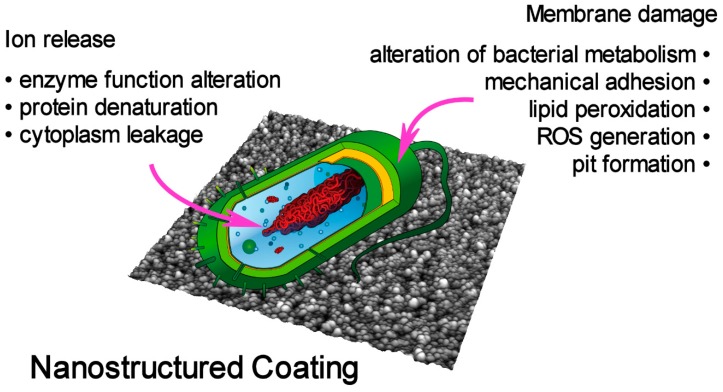
Schematic representation of a bacterium on a surface of a nanostructured coating. The major coating/bacterium interaction mechanisms are listed.

The chemical elements composing the NPs determine the mode and the efficacy of bactericidal action. Hence, to devise antibacterial nanostructured coatings, it is important to briefly review some of the possible nanostructured coating interaction mechanisms with bacteria, which are summarized in Figure 1. The scheme is based on the current knowledge of the interaction of NPs with the cell barrier and with the cytoplasmic components. Most of the results reported below, if not all, were obtained for NPs dispersed in the solution containing the bacteria, so some care should be taken to directly explain the bactericidal effect of a nanostructured coating by applying the same processes. 

### 2.1. Cell–NMs Interaction 

#### 2.1.1. Membrane Damage

Due to the different composition of the cell barriers of GP and GN bacteria, the interaction pathways with NMs vary from strain to strain, and therefore the antimicrobial action will depend on the specific composition, shape and chemical state of the NPs forming the NMs. As a general trend, the outer membrane of GN bacteria possess lipoproteins and phospholipids that form a penetration barrier allowing the entrance of macromolecules only, resulting in a greater NPs activity against GP bacteria than against GN bacteria [25,70,72]. Table 1 summarizes the interaction mechanism described below.

*Mechanical Adhesion.* In the bacteria–NPs interaction, one fundamental issue is the proximity or contact of the NPs with the outer bacteria membrane. If the NPs are dispersed in a solution, the efficacy of the antibacterial activity could be limited by the lipopolysaccharides present in the GN bacteria wall. Such molecules may prevent the adhesion of NPs to the cell barrier, and even regulate the exchange flow of ions through the bacterial cell membrane, thus reducing the killing effect of the NPs [40,73]. However, NPs are able to interact with the bacterial cell membrane, since the NP surface atoms can also bind with the negatively charged carboxyl and phosphate groups present in the bacterial cell membrane [25,40,74].

*Lipid peroxidation.* It has been found that ZnO NPs generate strong lipid oxidative decomposition through the yield of H_2_O_2_, causing the leakage of intracellular contents and bacteria death [73]. A different reaction pathway is provided by Al_2_O_3_ NPs. Attenuated total reflection data showed that such NPs interact with lipopolysaccharide through hydrogen binding and ligand exchange. Structural changes in the phospholipid lead to the destruction of the cell membrane and cytoplasmic leakage [75].

*Alteration of bacterial metabolism.* In the case of nanodiamonds with different oxygen-containing surface groups, it is the formation of covalent bonds with proteins and molecules on cell walls to induce a disorder of the bacterial metabolism and, finally, cell death [76], while the exposure to Fe and Cu NPs of two GP and GN bacteria species results in the hindering of the normal metabolic pathways [77].

*Reactive ion species (ROS) generation.* Apart from direct interaction through the chemical bond formation related to the surface composition of the NPs, other pathways are provided through the NPs’ physical properties. Since the outer membrane of the bacteria is charged, the interaction with oxidizing species has been recognized as an important antibacterial mechanism of NPs. The interaction of atoms with a strong positive redox potential with the bacteria wall induces an oxidative stress, leading to bacteria killing [73,78,79,80]. Depending on the material, shape and size of the NPs, different ROS can be produced by reducing oxygen molecules. CaO and MgO can produce superoxide radicals (O_2_^−^), ZnO might generate hydrogen peroxide (H_2_O_2_) and OH, while CuO NPs can also give rise to oxygen [40]. The generation of ROS can be obtained by photon absorption, employing the availability of surface states and defects to uptake the photogenerated electron hole pair to the surface of the NPs [81,82]. The absorbed photon generates and electron-hole pair that, once it has reached the surface of the NP, can interact with molecules adsorbed on the NP surface [83]. For instance, on ZnO the holes on the surface can produce hydroxyl radical OH by interaction with OH^−^, and the superoxide radical (O_2_^−^) by employing the photogenerated electron interaction with O_2_. 

*Pit formation.* TiO_2_ NPs adhered to the surface of bacterial cells produces ROS due to the photocatalytic action of the surface Ti atoms, which makes electrons available at the surface of the NP. The composition and structure of the cell membrane is damaged by the negatively charged Oxygen atoms, causing pit formation, leakage of cellular contents and subsequent bacterial death [84]. Cytoplasmic leakage also results from the increase in bacterial cell volume and oxidative decomposition of the bacterial membrane induced by the photocatalytic activation of TiO_2_ NP adhered to cell membrane [85]. Ag NPs have also been shown to be incorporated into the bacteria membrane, thus inducing a formation of pits and therefore the bacterial death [86].

Although TiO_2_ has been used most extensively because of its catalytic properties, economic feasibility, and enhanced stability, a lot of research has been pursued to overcome the major limitations of this material, i.e., the large bandgap (3.2 eV), which does not allow the use of visible light to produce electron hole pairs [6,7,87,88,89,90] and the high recombination rate of the e-h pairs [80,88,91]. Even if repeated experiments have shown that TiO_2_-based materials are more efficient in generating ROS species than ZnO-based materials [92], the application of doped TiO_2_ with increased light absorption in the visible range to antibacterial coatings is basically unexplored.

#### 2.1.2. Ion Release

NPs are able to release metal ions into the media surrounding the bacteria, hence giving rise to different effects depending on the interaction pathways of the ions.

*Protein denaturation.* A double effect of Ag+ ions interacting with the bacteria was proposed, namely: a) the ions denature the proteins of the bacteria, inducing the condensation of DNA molecules and hence their replication ability; b) Ag ions interact with the thiol groups in protein inducing their inactivation [93]. Ag protein interaction has also been proposed as a general way to alter the bacteria functionality since it is interacting with many different proteins [94].

*Cytoplasm leakage.* TiO_2_ NPs were found to induce a strong reduction of the membrane potential, resulting in the diffusion of the NP-generated hydroxyl radicals into the bacteria and in the leakage of cytoplasm [95], while a similar effect resulting from exposure of *E. coli* cells to ZnO NPs was attributed mainly to the ion/membrane interaction [96].

*Enzyme function alteration*. A recent work highlighted the effect of CuO NPs on the nitrogen metabolism and on the electron transfer on bacterial denitrification, by causing a significant alteration of the expression of key proteins. The exposure to CuO NPs affected the bacterial membrane integrity resulting in membrane damage, but also resulted in the inhibition of the glucose transport, thus affecting the bacterial intracellular metabolism [97].

### 2.2. Antibiofilm Activity

The surfaces of artificially implanted devices are conducive to the proliferation of eukaryotic pathogens, showing up as biofilm formation, hence becoming resistant to antimicrobial agents. From the point of view of NM design, one of the key steps is the adhesion of pathogen cells to the substrate. It has been suggested that this process starts with the action of adhesins, which act as specific surface recognition molecules [36], and calls for a specific understanding of the NMs’ surface properties in order to prevent biofilm formation. In human implantable devices (heart valves or dental implants) with antimicrobial coatings, TiO_2_ coatings loaded with NP of Ag, Ca and Si have been shown not only to favor osseointegration [34], but also to prevent thrombosis and the occurrence of inflammation through the reduction of biofilm formation [33,34]. In the case of partially implantable devices, such as catheters, the reduction of infection risk has been investigated by coating the catheter surface with Zn-doped CuO NPs to retard the growth of biofilms [35] and reduce the risk of bacterial infection and complications [99]. The coated catheters present promising antibiofilm activity, biocompatibility, and absence of detectable irritation [35]. In dental implants, and particularly in root canal therapy, embedding nanodiamonds functionalized with amoxicillin, a broad-spectrum antibiotic, leads to improved mechanical properties of the filler and functionality of the drug [100]. In maxillofacial prostheses, the reduction of the incidence of tissue inflammation surrounding the prostheses has been explored by coating the surface with nanostructured TiO_2_. As a result, the introduction of nano-TiO_2_ coatings has been shown to inhibit a rather large range of bacteria strains [101]. These few examples suggest that the combination of different elements into the coating is likely needed to provide a wide spectrum of anti-biofilm activity, though the role of the coating roughness has not been explored in detail. On the other hand, investigations into the effect of individual NPs on the biofilm disruption has been obtained for Au [36,102], Ag [103], Mg [104], ZnO [42,102,105], CuO [102,106], CeO [102], Fe_3_O_4_ [107], YF [108] and TiO_2_ [101], indicating that smaller NPs with high surface to volume ratio have a remarkable effect on biofilm destruction [98].

These works led to the development of various approaches with direct applications to the biomedical field, such as tailored surfaces with antimicrobial effect, wound dressings and modified textiles [5]. The use of linens impregnated with CuO NPs has been shown to reduce the occurrence of hospital-acquired infections in health care facilities. Bed sheets containing CuO NPs are considered one of the most interesting innovations in medical care, since they reduce microbial attachment and thus microbial infections within hospitals [109]. 

Since the possible interaction mechanisms between a coating and bacteria are manifold, this overview suggests that there is still a wide potential for tailoring the coatings properties by modifying the composition, the surface morphology, the structure and crystallinity of the surface of the coating and of the NPs composing the film. In the next section we will review some of the most recent works on nanostructured coatings obtained by two industrially scalable physical methods, MS and SCBD, to provide hints on the pathway to use a combination of different elements to widen the bactericidal spectrum. Considering that a coating should also provide durability against usage, some aspect of the mechanical properties of coatings will also be considered. 

## 3. Nanostructured Coatings

The strategies developed to control the incidence of infections should depend on the applications which they are devised for, i.e., on the type of surface where the antimicrobial agent has to be located, since the topological and chemical characteristics of a surface determine the rate of microorganism adhesion [19,21,46,48]. In the framework of synthesis of nanostructured coatings with antimicrobial properties, the main explored strategies range from modification of surfaces through deposition of polymeric films [19,21,45,49,50,51] with incorporated bactericidal agents [21,52,53,54,55], or deposition of metallic or oxide films [2,21,56]. For instance, Ag can broaden the bactericidal activity of TiO_2_-based photocatalyst composite materials and can also act against silver-resistant microorganisms due to their photooxidative mechanism [57]. Such work highlights just one of the many critical issues to be faced in the design of a functional coating, and in the following we will bring few examples related to nanostructured coatings, namely the adhesion (related to film composition), the substrate temperature during deposition (some substrates cannot withstand deposition temperatures above a few tens of °C), the coating porosity and surface to volume ratio, the duration of the coating or of the antimicrobial effect and /or toxicity, related to the mechanism of bactericidal action.

### 3.1. Magnetron Sputtering

The numerous synthesis techniques available for the production of NMs can be broadly divided into wet and non-wet routes. As previously described in the introductory paragraph, a vast literature covers wet synthesis approaches, in particular for NPs synthesis and antimicrobial behavior. On the other hand, the studies on nanostructured coatings are far from being complete, likely due to the fact that the technological application and industrial scalability of physical deposition techniques are still an open issue. In this sense, MS is becoming a rather mature technique, and is now applied at the industrial level to produce hard coatings on tools or decorative treatment of surfaces.

**Figure 2:**
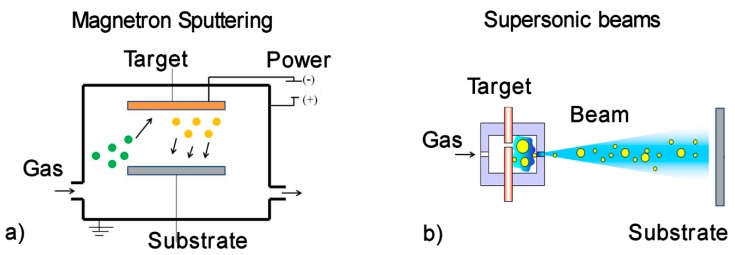
(**a**) Scheme of the magnetron sputtering process.; (**b**) scheme of the beam synthesis from pulsed gas sources.

Recently, the MS route [110] has been used also to deposit coatings on textiles and catheters [111], implants [112] and on food packaging [2,113]. At the laboratory level, this technique allows the synthesis of thin films up to the micron scale, together with the possibility of mixing materials to obtain the desired functionality [44,111,114]. In short, the sputtering process is obtained by applying an electric field between two electrodes within a medium vacuum chamber (see the scheme in Figure 2a). The gas (typically Ar) injected into the chamber is ionized by the electric field and bombards the target cathode, causing the ejection of atoms/molecules toward the substrates. The electric field may be applied in direct current mode, at radio or microwave frequency, or in pulsed mode, depending on the target conductivity and desired film control [115]. However, MS presents some limitations in depositing NPs with a controlled surface-to-volume ratio, and even more when core shell or Janus-like NPs might be needed to expose the active material in a controlled way. In particular, substrate temperature during deposition and resulting morphology of the film are two critical issues impacting the behavior of the film.

**Figure 3:**
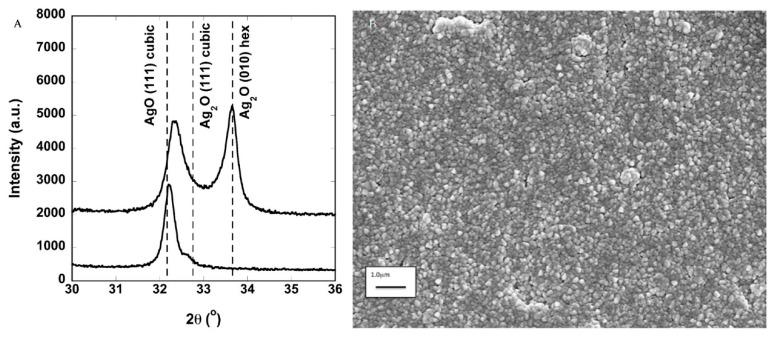
(**A**) Plot of the X-ray diffraction intensity versus 2θ showing single phase cubic AgO and mixed phase AgO and Ag_2_O deposited at lower oxygen partial pressure; (**B**) scanning electron micrograph showing the typical surface microstructure of the silver oxide deposited at room temperature. The microstructure can be impacted by deposition pressure, deposition power, oxygen partial pressure, and coating thickness. Reprinted from [67] under the Creative Commons Attribution License 4.0.

Goderecci et al. [67] synthesized 150 nm thick AgO/Ag_2_O films on sapphire reporting on Ag ion dissolution/elution rate, and bactericidal efficacy. The coatings obtained are polycrystalline films with an average grain size of around 100 nm, and can be grown with a single AgO phase or mixed AgO/Ag_2_O phases, as evidenced in Figure 3. 

The Ag ions elution under dynamic fluid flow ranges between 0.003 and 0.07 ppm/min, with a lower rate for complex cell culture media [67]. This rate is probably the cause of the good bactericidal activity obtained by these films that are composed of pure AgO. In this respect, the purity of the material and its concentration at the surface determine the bactericidal activity through the continuous release of Ag ions from the oxidized AgO phase. This suggest including the mechanism of action of this coating obtained by MS within the “ion release” group (see Figure 1), although there are no data to discriminate within the subset of different effects related to the metal ion interaction with the bacteria. Moreover, one cannot exclude a contribution to the antibacterial activity resulting from the membrane damage. Finally, the continuous release of Ag ions results in an increasing concentration of the element into the media in contact with the film, eventually inducing toxicity effects against mammalian cells. This points out the critical problem of dosing the amount of the bactericidal element to obtain a balance between antimicrobial action and toxicity side effects. This fact also impacts the applicability of this peculiar coating. The data reported by Goderecci et al. suggest that in a closed system, the accumulation of Ag ions will lead to toxicity, while this is likely to be avoided in an open system, where released Ag ions can be transported away. Hence, this kind of coating on a permanent implant that remains surrounded by living cells could not be applicable. On the other hand, one could envisage the use of such films in a wastewater purification system where the liquid flow is continuous.

It is interesting to note that such films composed of a single element present good adhesion, as shown by the ASTM D3359 cellophane tape test on hard and flexible substrates. On the other hand, the mixing into the coating of elements needed to obtain killing activity against both GP and GN strains has raised the issue of the coating mechanical stability [44,56,116,117,118]. Musil et al. [44,118] suggested that in this kind of thin film, the relative content of the most efficient antibacterial metals, Ag and Cu, needs to be between 10% and 30%, and this almost always results in a strong reduction of its hardness and in a poor mechanical stability, in particular if the film thickness is on the order of hundreds of nm. This is a major drawback, since many practical applications of antibacterial coatings on contact surfaces of rigid or flexible substrates require a long lifetime, and therefore hardness and resistance to wear [56,116,117]. Moreover, when such coatings are deposited on flexible substrates, they easily crack and/or delaminate due to the residual stress resulting from the growth mode (for some reviews on thin film growth modes and on the effect on surfaces, see for instance [119,120]).

**Figure 4:**
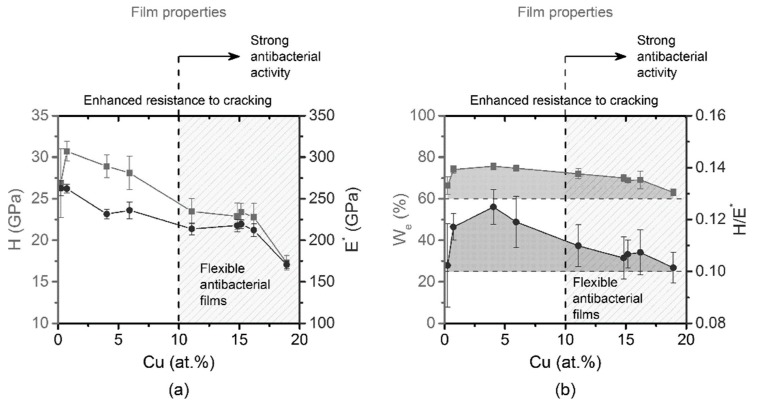
(**a**) Hardness H (gray squares), effective Young’s modulus E* (black circles); and (**b**) elastic recovery We (gray squares) and H/E* ratio (black circles) of Zr–Cu–N coatings sputtered on Si (100) substrates as a function of Cu content. Reprinted from [121], Copyright 2015, with permission from AIP Publishing LLC.

This issue has recently been investigated for antibacterial films prepared by reactive magnetron sputtering for Cr-Cu-O [44,118], Al-Cu-N [44,114] and Zr-Cu-N [121], where the influence of Cu content on the mechanical and bactericidal properties of the film have been measured. The mechanical characteristics measured through Vickers tests were the film hardness H, defined as its resistance to local plastic deformation [122], the elastic recovery We, defined as the fraction of a given deformation of a solid which behaves elastically [123], the Young’s modulus E and the effective Young’s modulus E* = E(1 − ν^2^), where ν = Poisson ratio has been obtained by mechanical indentation. The results obtained on the Zr–Cu–N coatings are summarized in Figure 4a,b, where the mechanical properties are plotted as a function of Cu content in the Zr–Cu–N coating.

The hardness H and the effective Young’s modulus E* decrease with increasing Cu content, while the elastic recovery We is ≥ 60%. The Zr–Cu–N coatings exhibit a ratio H/E* ≥ 0.1, a value indicating an enhanced resistance to cracking for all Cu contents ranging from 0 to 19 at.% Cu. [121] Moreover, the hardness H ranging from ~25 to ~17 GPa is quite high and it makes it possible to prevent the coating from being removed from the surface of a substrate by fretting (wear). The region of Cu content in which the coatings exhibit a killing of the *E. coli* bacteria of 100% is marked by dashed lines and in light gray, setting the limit for the minimum Cu concentration in such films [121]. The good mechanical properties obtained for such films have been obtained thanks to a deposition substrate temperature of 450 °C, and this might be a major drawback when the same behavior is needed to form films on flexible substrates that would melt at that temperature. Moreover, in the investigated films, the active surface is limited to the top plane, resulting in a very small surface-to-volume ratio as compared to those of film nanostructures at the 10 nm scale. Finally, the use of precious metals like Ag to widen the bactericidal spectrum could result in an increase of the production costs, considering the total amount of the bactericidal metal needed to obtain a micron thick material over cm^2^ areas. Unfortunately, the reported results do not indicate the mechanism of action of the synthesized coating and concentrate on the physical properties of the film, so at this stage one may hypothesize that the antibacterial activity might come from both ion release and membrane damage.

### 3.2. Gas Phase Deposition

A feasible alternative to tackle the different issues discussed above is to employ a different physical method, namely Supersonic Cluster Beam Deposition (SCBD) [6,70,71,124,125,126,127]. The peculiar characteristics of this method are related to the working principle described in detail elsewhere [19,90,126,127] and schematized in Figure 2b. Briefly, an inert gas (He or Ar) is injected into an ablation chamber at pressures in the 20 to 45 bar range towards a target rod of the desired material. The carrier gas allows the triggering of a synchronized high voltage discharge, thus forming a plasma that ablates the Ag rod. The ablated material condenses into NPS that are extracted through a nozzle to reach supersonic speed in the expansion chamber, thus forming a collimated beam directed on the substrate surface. Considering the fact that the NPs are produced in gas phase under controlled pressure conditions and deposited in medium vacuum, the setup allows the deposition of nanostructured film on virtually any substrate, either rigid or flexible [12], without the presence of solvents or other contaminants. The average kinetic energy of the NPs (about 0.2 eV/atom) [3,127] allows a soft landing of the NPs that maintains their original structure, hence NPs are assembling on the substrate forming a film of desired thickness. It is also worth noting that the coating is assembled from NPs landing and the deposition takes places at room temperature, hence avoiding the risk of substrate damage.

**Figure 5:**
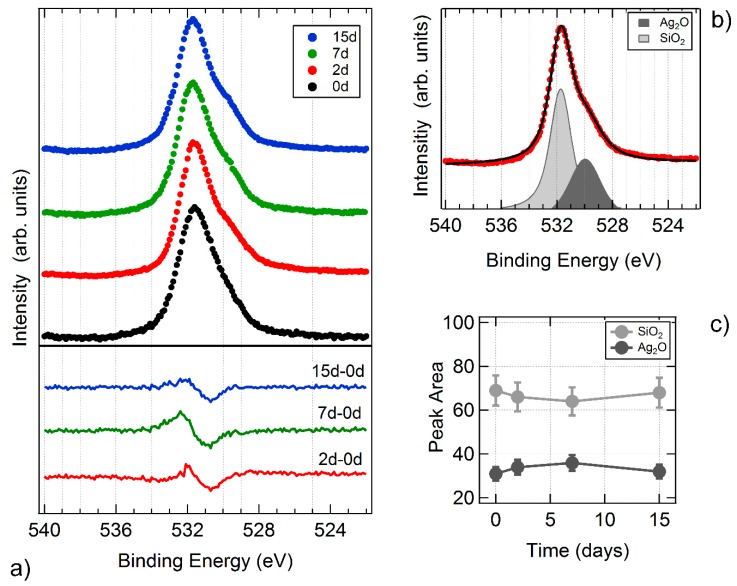
(**a**) Normalized O1s core level spectra obtained from the as-deposited Ag NPs film (curve 0d) and from the same film two days (curve 2d), seven days (curve 7d) and fifteen days (curve 15d) after the deposition. In the bottom panel the difference spectra show that the variation of the peak observed after 2 days (curve 2d-0d) remains mostly unchanged up to 15 days. (**b**) O1s core level obtained from the Ag NPs film two days after deposition, with the peaks resulting from the least square fitting procedure. The AgO related peak (dark gray) is at 530.2 eV binding energy while the SiO_2_ related peak (light gray) is at 531.6 eV binding energy. (**c**) intensity dependence of the relative area of the two peaks as a function of time.

SCBD has been used to synthesize antibacterial coatings only in recent years [70,71]. The pioneering work of Cavaliere et al. [70] investigated the nanostructured Ag films obtained by SCBD and their bactericidal activity. The films are deposited at room temperature on soda lime glass substrates. The average NPs size was 7.8 ± 0.6 nm, and the correspondent film thickness was measured to 8 nm, i.e., a single layer, with a NP density of 1.15 ± 0.04 × 10^11^ NPs/cm^2^ [70]. The film is stable against ageing up to 15 days, as can be deduced from Figure 5, where the X-ray photoemission spectra of the O1s core level are plotted as a function of time. Very small changes are observed in the oxygen line shape as a function of time, as evidenced by the difference lines on the bottom of the panel. A least square fitting procedure decomposes the O1s peak in a main component at 531,6 eV binding energy, related to the Si-O bond in the substrate [128], and to the AgO feature at 530.2 binding energy [128,129] present in the film. The relative intensity of the two components as a function of time shown in Figure 5c show a maximum change of 10% with respect to the initial value, indicating major stability of the film against variation of the oxidation state.

As for the mechanical properties, coating thicknesses below 10 nm, as that obtained in the work of Cavaliere et al., hamper the possibility to obtain information on the mechanical characteristics of the system with standard indentation techniques, although recent attempts to unveil the hardness, elastic modulus, adhesion and friction of single NPs have been reviewed [130]. Modelling of atomic force microscopy interaction with a single nanoobject is actually well established, although experimentally very little data on the NPs/substrate interaction obtained by this technique is available [130]. The same technique, however, might not be applicable when the grown film is granular or porous, i.e., the NPs are assembled into a film while conserving their structural individuality, thus making the film subject to initial plastic modifications under the load of the AFM tip, hampering a reliable extrapolation of the elastic constant from force distance curves.

To overcome this problem, a recent study on nanoporous granular film was carried out using the picosecond photoacoustic technique [131,132], an optical technique that probes the mechanical behavior of granular materials maintaining their original morphology and properties. The picosecond photoacoustic technique is based on the excitation of the mechanical breathing automodes of the film by a 150 fs laser pulse. The excited elastic eigenmodes are then sampled measuring the variation in the reflectivity or transmissivity of a time-delayed probe pulse. The laser spot size is around 100 microns against a film thickness in the order of tens of nm, hence, the only eigenmodes that are excited/sampled, are those with a displacement field perpendicular to the film surface. The film may be considered as a *homogenous effective medium*. Its elastic properties are thus described by two elastic coefficients, namely c_11_ and c_44._ The data provide the elastic eigenmodes period and decay time. With these at hand a simple continuum mechanical model makes it possible to determine the film elastic constant c_11_ and yield information on the film adhesion to the substrate [131]. For the case of pure Ag NPs films, the density, longitudinal sound velocity, and perpendicular elastic stiffness (c_11_) are 80% and 50% of the respective values for bulk polycrystalline Ag, that is rather lower than typical values obtained for coatings deposited by magnetron sputtering.

**Figure 6:**
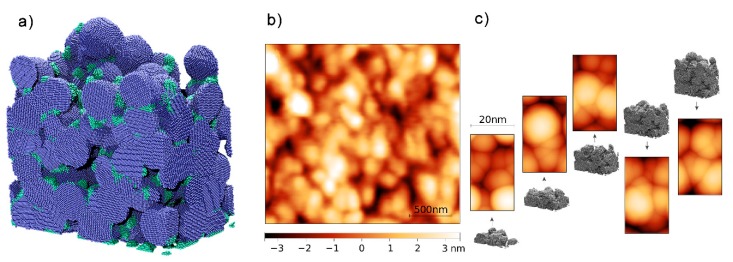
(**a**) The NPs virtual thin film (dimensions L_X_ × L_Y_ × L_Z_ = 35 nm × 20 nm × 30 nm) obtained by MD simulations. The NPs are divided into blue (large, diameter ~ 6 nm) and green (small, diameter ~ 1 nm). (**b**) Experimental AFM image of the 30 nm-thick Ag NPs film. (**c**) Computed AFM images obtained from the simulated cell and taking into account tip convolution effects. The computed images are obtained from intermediate deposition steps of the MD simulations, i.e., subsequent shots of the simulation resulting in films of average thickness ⟨t_F_⟩ = 9, 14, 23, 27, and 31 nm for shots one through five, respectively. Adapted from [132] (https://pubs.acs.org/doi/10.1021/acs.jpcc.7b05795), with permission from ACS (further permissions related to the material excerpted should be directed to the ACS).

Such results are consistent with the porous nanostructure of the film obtained by molecular dynamics (MD) simulations [132]. In particular, the intrinsic porosity of the coatings obtained by SCBD can be appreciated from the virtual film reported in Figure 6a. The calculated structure provides elastic properties and surface morphology well in agreement with the experimental data [132], and suggests that the low residual stress of the film and the estimated adhesion to a sapphire substrate are directly related to the granularity of the film.

**Figure 7:**
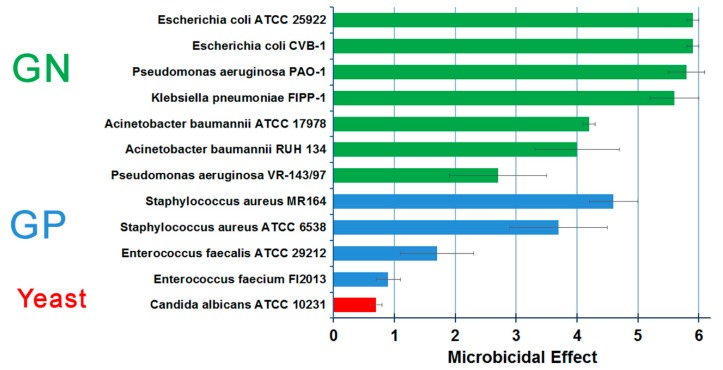
Quantification of the ME for different extensively drug-resistant phenotypes. All microorganisms were tested in three independent experiments and results were averaged. To calculate standard deviations (SD), when no viable cells were counted, the result was arbitrarily assumed as 4.2 × 10^1^ CFU, representing the detection limit value.

The Ag NP films investigated in the work of Cavaliere et al. [70] were found to exert a broad-spectrum bactericidal activity, quantified by depositing a 10 µL of microorganism suspension (i.e., range 5.4–7.3 log Colony Forming Units—CFU) both in a glass slide covered with the Ag NPs film and in a control slide. After 24 hours of incubation at 25 °C in protected damp environment, microorganisms were suspended in 10 mL of PBS, and 240 µL of each dilution was plated for viable cell count. The microbicidal effect (ME) was defined as ME = logNC − logNE, where NC and NE are the CFU obtained with control slides and NP film slides, respectively. The ME was demonstrated both with reference strains and with a collection of clinical strains that exhibited extensively drug-resistant phenotypes and/or belonged to high-risk hyperepidemic clones, in particular clinical strains producing two of the most worrisome resistance mechanisms recently emerged in enterobacteria and capable of pandemic dissemination [133]. The obtained results are summarized in Figure 7. Some of the sterilized microorganisms are major opportunistic pathogens in the hospital setting, with a high propensity to survive for long periods on surfaces of hospital environments, evidencing the need for a strategy to reduce the cross-contamination epidemics. The results obtained in the work of Cavaliere et al. [70] indicate that the film, being composed of Ag and AgO, has a limited efficacy on the film on some GP bacteria. It has been hypothesized that such behavior could be due to the overall reduction of anionic surface charge, associated with daptomycin resistance in S. aureus [134].

This would suggest a bactericidal mechanism similar to the ion release observed in [67], although the film morphology and thickness are very different, in particular the average size of the NPs differs at least by an order of magnitude. 

**Figure 8:**
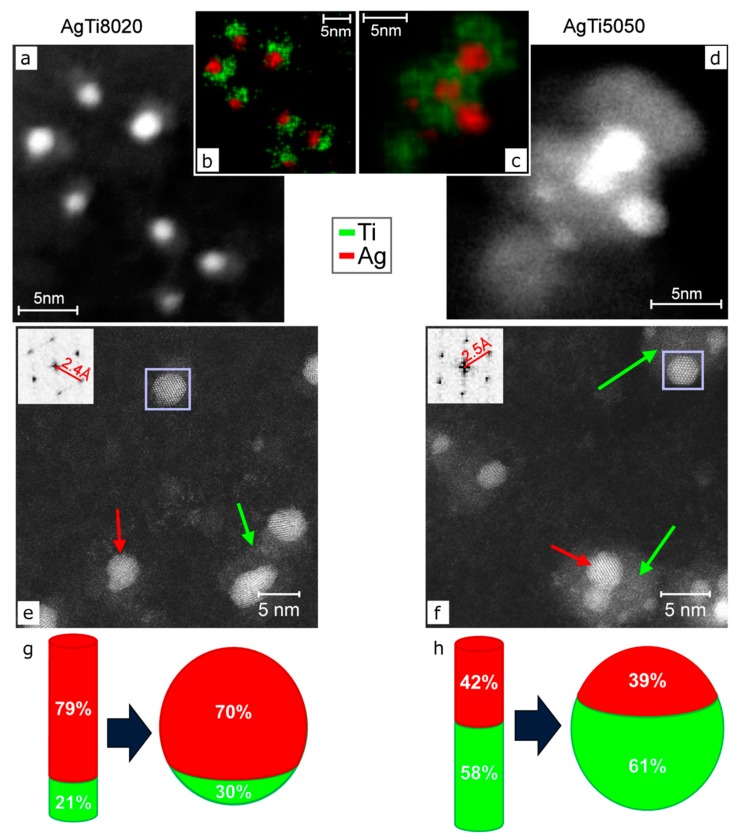
(**a**,**d**) TEM images of AgTi8020 and AgTi5050 scattered NPs, respectively, with the relative elemental map plotted in panels (**b**,**c**), respectively. The data show that Ag and Ti are phase-separated into the NPs. (**e**,**f**) HR-STEM images of the NPs, with the inset showing the FFT analysis of Ag crystalline structure of the zone in the purple rectangle. Red arrows indicate small Ag NPs, and green arrows point to the Ti part of the NPs. The data indicate that Ag is crystalline and Ti is amorphous. (**g**,**h**) Schematic representation of the elemental weight in the initial rod and in the NPS, showing the good correspondence of the material concentration. Adapted from Ref. [71] under the Creative Commons Attribution License 4.0.

Despite the indication that the pure Ag nanogranular film presents a rather good adhesion to a sapphire substrate, further work has been carried on with the goals of reducing the amount of precious metal, widening the bactericidal spectrum and further increase the film adhesion. This route was pursued again using SCBD, since this technique has been employed to grow NPs films of different materials (Ti [3,90], C [135], Pd [136]) but has also been used to dope TiO_2_ NPs with Cr and N at atomic concentrations of 3% to 7 % [6,7,8,71,124], suggesting that the method could in principle be able to deposit NPs and granular films with tunable element concentrations. The flexibility of the methods to directly synthesize and deposit coatings with multi-elemental NPs has been demonstrated in the work of Benetti et al., showing the possibility to synthesize bi-element NPs of Ag and Ti with variable elemental concentrations [71,125]. Two different rods for the SCBD setup have been used; the first (AgTi8020) has nominal weight contents of 80% Ag and 20% Ti, and the second (AgTi5050) has nominal weight contents of 50% Ag and 50% Ti [71,125]. The synthesized NPs, characterized by high-resolution scanning transmission electron microscope (HR-STEM), can be observed in Figure 8. The intense spot partly surrounded by a lighter gray zone (Figure 8a,d) find an explanation in the corresponding elemental maps (Figure 8b,c): the contrast difference is because Ag (shown in red) and Ti (shown in green) are phase-separated into the NPs. The detailed investigation of the structural and chemical composition of the NPs [71] indicate that such bi-NPs are composed of Ag nanocrystals intermixed and embedded into amorphous Ti. This NPs structure [137,138] is remarkably different from the simple Ag decoration of TiO_2_ nanostructures obtained by sol–gel [57], solvothermal [58], hydrothermal [59], and hydrolyzed solution [139] methods. Moreover, the work clearly indicates that the compositional changes in the pristine rod (i.e., pure Ag, AgTi8020, AgTi5050) correspond to an analogous variation in the synthesized bi-NPs stoichiometry [71,125], proving that SCBD is a powerful tool for tailoring the NPs’ chemical composition by simply adjusting the Ag/Ti relative concentration in the initial rod.

Finally, the nanostructured Ag-Ti coating shows good adhesion to the substrate and exhibits an exceptional bactericidal effect against major GN nosocomial pathogens, remarkably similar to a pure Ag NPs coating [70], but with an 85% lower Ag mass content. The latter consideration points toward a favorable strong reduction of the total Ag content in an antibacterial coating, but also reveals that a partial Ag inclusion in a protective matrix such as TiO_2_ would not hamper the efficacy of the active metal. In turn, such behavior would again suggest a bactericidal mechanism related to the ion release schematized in Figure 1. As a future development, it would be worth exploring the bacteria/nanogranular film interaction to determine the major killing mechanism of such systems. 

The remarkable activities against GN bacteria of Ag [70,140] and against GP bacteria and yeasts of Cu suggests that the combination of these two elements widens the microbicidal spectrum of a coating [141,142]. However, one should keep in mind that the coatings should maintain good adhesion and stability against wear, and hence the presence of an adhesion promoter in the film is mandatory. Taking into account the negligible bactericidal activity of TiO_2_ in dark conditions, we opted to use Mg, an element with good microbicidal activity [143] and an oxygen chemical affinity higher than that of Ti [144]. Furthermore, the choice of using Mg as an adhesion mediator has been promoted by recent outcomes showing that its microbicidal activity is strongly enhanced by the presence of small quantities of Ag [145].

**Figure 9:**
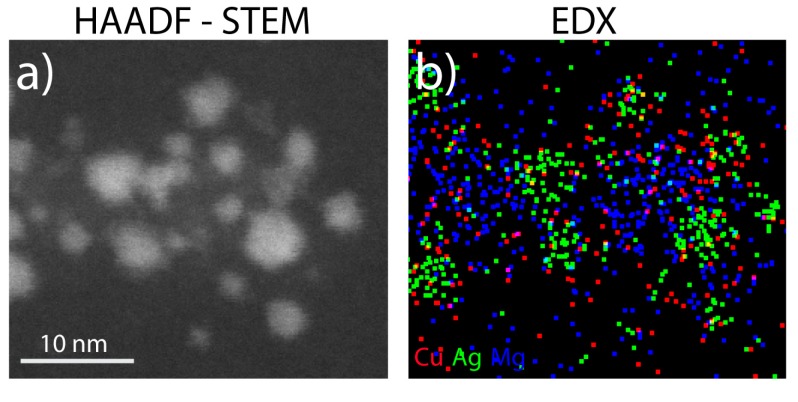
STEM (**a**) and corresponding EDX elemental maps (**b**) for the Mg/Ag/Cu NPs. Scale bar is 20 nm. Adapted from Ref. [146] under the Creative Commons Attribution License 4.0.

The material used as ablation target is a sintered rod composed of Mg, Ag and Cu at nominal concentrations 20/50/30% in weight. The first results of direct multi-element NP synthesis obtained by SCBD and deposited on TEM grids are exemplified in Figure 9 [146]. In the STEM image of Figure 9a, the heavier Ag and Cu appear as bright zones whereas Mg is visible as less bright areas of the nanocomposite system. The energy dispersive X-ray spectroscopy (EDX) map (Figure 9b) shows that the NPs assume a cluster-in-cluster form, with a matrix of Mg (blue in Figure 9b) partially embedding Ag (green) and Cu (red) clusters.

The EDX and X-ray photoemission spectroscopy elemental analysis suggests that Ag and Cu are metallic, while Mg is oxidized to MgO. Rutherford backscattering spectrometry shows that the relative weight content of the NPs (Mg/Ag/Cu = 24/47/29) is compatible with the nominal one of the starting rod (20/50/30), indicating a direct transfer of all the elements from the macroscopic rod to the nanostructured film. This direct correspondence demonstrates the possibility to finely tune the elemental composition of tri- and multi-element nanoparticles.

**Figure 10:**
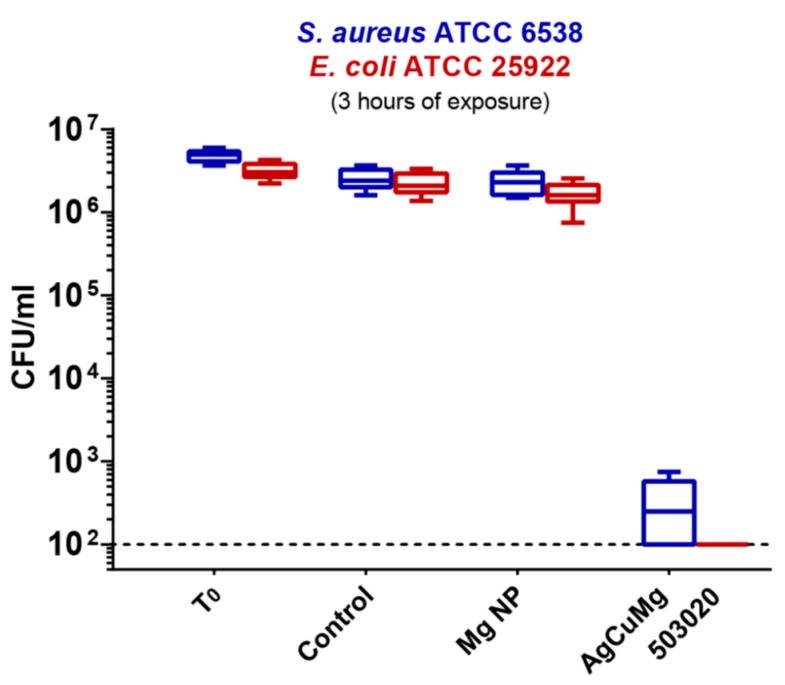
Microbicidal tests on S. aureus (blue) and E. coli (red), comparing the count of viable bacteria (reported as CFU per milliliter) of the control before incubation (T0), control bare substrate after incubation (Control), pure Mg NPs (Mg NP), and tri-elemental AgCuMg503020 film. The dashed line at 10^2^ CFU ml^−1^ is the limit of detection of the experiment. Reproduced from [146] by permission of the PCCP Owner Societies.

In view of the NP film deployment as a wide-spectrum antibacterial coating, knowledge of the actual Cu and Ag distribution within the NPs is indeed desirable. Exposure of both Cu and Ag is a matter of fact beneficial to attack both GP and GN strains. Inspection of Figure 9b does not make it possible to experimentally distinguish the distribution of Ag and Cu, except for the fact that they are both located in the metallic part of the NPs. By taking into account the immiscible nature of Ag and Cu [147], one could expect a the segregation of these two elements instead of an alloyed core. MD simulations reveals that the NP structure is characterized by a partial Ag core surrounded by phase-segregated Cu at the interface with MGO [146], a chemical ordering opposed to the typical Cu_core_Ag_shell_ arrangement for AgCu NPs in the gas phase [148,149,150]. The antibacterial activity of such tri-element nanogranular film against drug-resistant GN and GP reference strains (*Escherichia coli* ATCC 25922 and *Staphylococcus aureus* ATCC 6538) has been quantified using the procedure employed for AgTi NPs [71] and is shown in Figure 10. The striking sterilization of the samples observed for both strains is likely related with the presence of exposed Cu and Ag, since it was not observed in the case of pure Mg NPs. Such behavior might be consistent with the hypothesis of ion release from the NPs as a mechanism of bacteria death, although one cannot exclude other pathways as mechanical adhesion to the coating and enzyme function alteration, as reported for the NPs cases summarized in Table 1. 

The efficacy of antibacterial coatings obtained by SCBD and MS is summarized in Table 2. For the nanogranular film obtained by cluster beams it is possible to quantify the ME (indicated by the number of dots in the table). In the reported cases where MS has been employed, the ME is defined with a different methodology and hence the simple presence of the effect is indicated. The summary reveals that for the MS case many data on the ME for both GN and GP strains are missing, calling for a thorough investigation in the future.

It should be also noted that for the coatings discussed in the present review, there are no data directly reporting on the mechanism leading to bacteria death, except for the work of Goderecci et al. [67], where the Ag ions release by the coating has been suggested as the cause of the bacteria death. The fact that the NPs are assembled into a coating, which determines a collective behavior in term of physical properties (e.g., optical absorption, roughness, stability, wettability ion diffusion across the coating), might influence the killing mechanism. Revealing how these innovative coatings lead to the microbicidal effect will make it possible to design nanostructured coatings for each specific applications.

## 4. Summary and Perspectives

Nanostructured coatings synthesized by physical methodsare emerging as a vaiable solution to limit the spread of bacteria in different environments, in particular in health-related settings where contamination of medical device surfaces is a major issue. The absence of solvents in the synthesis process allows for superior control over the material purity, film, thickness, relative concentration of the elements composing the coating, and, furthermore, the possibility of modifying the mechanical properties. The fabrication of coatings by techniques such as magnetron sputtering has already reached the industrial level, the same cannot yet be said for SCBD despite its advantages. SCBD allows direct deposition of the coatings in a nanostructured form and extreme flexibility in the choice of materials, allowing to tailor the coating for each specific application. The coatings can in some cases be fabricated with good hardness and resistance to wear, and the adhesion to the substrate can be increased by using materials such as TiO_2_ or MgO. A subject yet to be thoroughly investigated is the determination of the antimicrobial activity mechanism, which requires, however, a strong interdisciplinary effort.

A few considerations can be made to address the future perspectives and challenges in the field. The first issue for antibacterial surfaces is its long-term stability and the capability of a surface to maintain its properties over a time scale of days, week, months, or even years. The life-time of an antibacterial surfaces is, of course, strongly dependent on the application. For instance, on medical implants the antibacterial activity duration could be restricted to the period required by the body to accept the implant, while on medical devices used in surgery, the antibacterial effect could be restricted to a few hours. Hence, despite the successful killing of initially surface adhered bacteria over short periods, the efficacy over a time longer than a few hours after usage is still an open issue. This also raises the question as to what is the most important bactericidal mechanism of the films: ion release, ROS generation or surface morphology? This should be thoroughly addressed in the future and this knowledge exploited to tailor the synthesis of nanostructured coatings. 

Another issue is related to antibiofouling activity of the coatings. A wealth of research has been performed, but mostly on paints. For this reason there is basically no information on the behavior of the described coatings with respect to biofilm formation. The third issue is related to the use of standard strains to test the bactericidal activity. This conduct does not take into account the emergence of antibiotic resistant strains, against which novel coatings should be tested. 

Finally, many of the reports to date are proofs of concept on some model surfaces, although both magnetron sputtering and SCBD are already simple, cost-effective, environmentally friendly, and reproducible fabrication methods. In this sense the industrial scale up should be envisaged in the near future. Tackling these important challenges will require collaborative efforts from researchers in the fields of surface chemistry, materials science, biomedical engineering, and biotechnology. The technological fall-out will also provide plenty of opportunities for innovation beyond antibacterial surfaces, like for instance in nonlinear optics materials, sensors or membranes.

## Figures and Tables

**Table 1:** Summary of the cell/NPs interaction mechanisms for which the NPs’ effects have been described.

Mechanism Leading to Membrane Damage	Mechanical Adhesion	Lipid Peroxidation	Alteration of Bacterial Metabolism	ROS Generation	Pit Formation
NP material	Ag [25]; MgO [72]; TiO_2_ [74];	ZnO [73] Al_2_O_3_ [75]	Nanodiamonds [76]; Fe, Cu [77]	CaO, MgO [40]; ZnO [78,92]; CuO [78]; SiO [98]; TiO_2_ [80,92]; GO [79]	TiO_2_ [85]; TiO_2_/Ag, TiO_2_/CuO [84]
Mechanism related to ion release	Protein denaturation	Cytoplasm leakage	Enzyme function alteration		
NP material	Ag [93,94]	TiO_2_ [95]; ZNO [96]	CuO [97]		

**Table 2:** Summary of the described coatings obtained by gas phase deposition and magnetron sputtering. For the SCBD films, the number of points represent an average of the microbicidal effect on the tested strains in the different works (1 dot corresponds to ME = 1). For the magnetron sputtering films, the ME is not defined in a consistent way, and therefore cannot be compared to the other sets of works and the *E. coli* strain tested is not specified, so the open square (☐) indicates that the coating has some antibacterial properties.

		Nanogranular Films by SCBD			Films by Magnetron Sputtering			
		Ag	AgTi	AgCuMg	Ag, AgO	Cr-Cu-O	Al-Cu-N	Zr-Cu-N
	Ref.	[70]	[71]	[146]	[67]	[118]	[114]	[121]
Effective against	GN	●●●●●	●●●●●	●●●●●	☐	☐	☐	☐
	GP	●●	●●	●●●●●	☐	NA	NA	NA
